# Hyphal differentiation induced via a DNA damage checkpoint-dependent pathway engaged in crosstalk with nutrient stress signaling in *Schizosaccharomyces japonicus*

**DOI:** 10.1007/s00294-012-0384-4

**Published:** 2012-10-23

**Authors:** Kanji Furuya, Hironori Niki

**Affiliations:** 1 Department of Genetics, Graduate University for Advanced Studies, Sokendai, Hayama, Miura District, Japan; 2 Microbial Genetics Laboratory, Genetic Strains Research Center, National Institute of Genetics, 1111 Yata, Mishima, Shizuoka, 411-8540 Japan; 3 Present Address: Department of Mutagenesis, Radiation Biology Center, Kyoto University, Kyoto, Japan

**Keywords:** Cell cycle, Dimorphism, Fission yeast

## Abstract

DNA damage response includes DNA repair, nucleotide metabolism and even a control of cell fates including differentiation, cell death pathway or some combination of these. The responses to DNA damage differ from species to species. Here we aim to delineate the checkpoint pathway in the dimorphic fission yeast *Schizosaccharomyces japonicus,* where DNA damage can trigger a differentiation pathway that is a switch from a bidirectional yeast growth mode to an apical hyphal growth mode, and the switching is regulated via a checkpoint kinase, Chk1. This Chk1-dependent switch to hyphal growth is activated with even low doses of agents that damage DNA; therefore, we reasoned that this switch may depend on other genes orthologous to the components of the classical *Sz. pombe* Chk1-dependent DNA checkpoint pathway. As an initial test of this hypothesis, we assessed the effects of mutations in *Sz. japonicus* orthologs of *Sz. pombe* checkpoint genes on this switch from bidirectional to hyphal growth. The same set of DNA checkpoint genes was confirmed in *Sz. japonicus*. We tested the effect of each DNA checkpoint mutants on hyphal differentiation by DNA damage. We found that the *Sz. japonicus* hyphal differentiation pathway was dependent on *Sz. japonicus* orthologs of *Sz. pombe* checkpoint genes—_SP_ *rad3*, _SP_ *rad26*, _SP_ *rad9*, _SP_ *rad1*, _SP_ *rad24,*_SP_ *rad25*, _SP_ *crb2*, and _SP_ *chk1*—that function in the DNA damage checkpoint pathway, but was not dependent on orthologs of two *Sz. pombe* genes—_SP_ *cds1* or _SP_ *mrc1*—that function in the DNA replication checkpoint pathway. These findings indicated that although the role of each component of the DNA damage checkpoint and DNA replication checkpoint is mostly same between the two fission yeasts, the DNA damage checkpoint was the only pathway that governed DNA damage-dependent hyphal growth. We also examined whether DNA damage checkpoint signaling engaged in functional crosstalk with other hyphal differentiation pathways because hyphal differentiation can also be triggered by nutritional stress. Here, we discovered genetic interactions that indicated that the cAMP pathway engaged in crosstalk with Chk1-dependent signaling.

**Electronic supplementary material:**

The online version of this article (doi:10.1007/s00294-012-0384-4) contains supplementary material, which is available to authorized users.

## Introduction

DNA damage causes cells to activate various molecular pathways and induces various cellular activities, including DNA damage repair, cell death, and even cellular differentiation (Carr 2002; Inomata et al. 2009; Wahl and Carr 2001). These responses are regulated or affected by DNA damage responsive (DDR) pathways, and one of these critical pathways is the DNA checkpoint pathway, which is a signaling cascade associated with intensive phosphorylation (Carr 2002). Proteins involved in this checkpoint pathway are evolutionally conserved among many eukaryotes, including between yeast and humans. The molecular functions and structures of these proteins were initially discovered via studies of yeast cells (al-Khodairy et al. 1994; Carr 2002; Weinert and Hartwell 1988). In the fission yeast (*Schizosaccharomyces pombe*), a central role of the DNA checkpoint response is carried out by the _SP_Rad3^ATR^–_SP_Rad26^ATRIP^ kinase complex (_SP_Rad3; human ATR {Ataxia Telangiectasia and Rad3 related} ortholog in *Sz. pombe*, _SP_Rad26; human ATRIP {ATR interacting protein} ortholog in *Sz. pombe*) which phosphorylates various DDR proteins as well as other checkpoint proteins (Carr 1997; Edwards et al. 1999; Enoch et al. 1992). Among the downstream components of this checkpoint pathway, either _SP_Cds1^CHK2^ or _SP_Chk1^CHK1^ is phosphorylated and activated by _SP_Rad3^ATR^ in response to a stalled DNA replication fork stall or damaged DNA structure, respectively (Lindsay et al. 1998; Murakami and Okayama 1995; Walworth and Bernards 1996). The activation of either of these effector kinases requires mediator proteins; specifically, activation of _SP_Cds1^CHK2^ requires _SP_Mrc1^Claspin^, and activation of _SP_Chk1^CHK1^ requires _SP_Crb2^53BP1^ (Alcasabas et al. 2001; Griffiths et al. 1995; Saka et al. 1997; Tanaka and Russell 2001). Furthermore, signaling between _SP_Rad3^ATR^ and effector kinases requires the _SP_Rad17^RAD17^–_SP_Rfc and the _SP_Rad9^RAD9^–_SP_Rad1^RAD1^–_SP_Hus1^HUS1^ (9-1-1) complexes and the _SP_Cut5^TOPBP1^ protein, which associates with Rad9^RAD9^, to play a key role as an activator of _SP_Rad3^ATR^ (Caspari et al. 2000; Furuya et al. 2004; Griffiths et al. 1995; Saka et al. 1997). There are actually slight differences in the configuration of the biological function of effector kinases in other organisms. In vertebrates, CHK1 is activated upon DNA replication fork stalling, and CHK2 is activated upon breakage of double-stranded DNA (Guo et al. 2000; Kumagai and Dunphy 2000; Matsuoka et al. 1998). In the budding yeast (*Saccharomyces cerevisiae*), the ortholog of _SP_Cds1^CHK2^ is _SC_Rad53; this *Sc. cerevisiae* protein is activated upon both DNA replication stress and DNA damage and is essential for most of the DNA checkpoint pathways (Allen et al. 1994; Weinert et al. 1994). Moreover, ATR, the vertebrate ortholog of *Sz. pombe*_SP_Rad3, differs in function from _SP_Rad3 because ATM has a major role in the response to double-stranded DNA breaks and because activation of ATM (AtaxiaTelangiectasia-mutated) leads to CHK2 activation (Matsuoka et al. 1998). In contrast, Tel1^ATM^, the ortholog of ATM in *Sz. pombe* and *Sc. cerevisiae*, has a minor role in activating effector kinases (Morrow et al. 1995; Naito et al. 1998). This difference between these yeast species and vertebrate species may be due to differences in the manner in which double-stranded DNA breaks are processed in these taxa because, in the yeasts, these breaks are quickly processed into single-stranded DNA. This newly formed single-stranded DNA would be immediately covered with single-stranded DNA binding protein RPA (Replication Protein A), which can accommodate ATR-ATRIP orthologs, and lead to the activation of the checkpoints (Zou and Elledge 2003).

Checkpoint activation prevents entry into M-phase, which is triggered by activation of Cdk (*C*yclin-*D*ependent *K*inase). Cdk sits downstream of the checkpoint pathway, and importantly, inhibitory phosphorylation on tyrosine 15 (Y15) of Cdk is the final target of the checkpoint cascade (Enoch et al. 1991). The regulation on Y15 phosphorylation is conducted by kinases and phosphatases that are placed downstream of the checkpoint pathway (Dunphy and Kumagai 1991; Featherstone and Russell 1991; Gould et al. 1990; Lundgren et al. 1991; Parker et al. 1991; Strausfeld et al. 1991). In case of *Sz. pombe*, the _SP_Wee1 and _SP_Mik1 kinases phosphorylate Y15, and Cdc25 de-phosphorylates Y15. Mik1, and Cdc25 are targeted either directly or indirectly by the effector-kinases _SP_Chk1^CHK1^ and _SP_Cds1^CHK2,^ although _SP_Wee1 is controversial for a role in checkpoint response (Christensen et al. 2000; Furnari et al. 1997; Raleigh and O’Connell 2000; Rhind and Russell 1997).

Checkpoint activation can also regulate the mode of cell proliferation. *Sz. japonicus* is a species of fission yeast. This species undergoes bidirectional growth and symmetrical division (yeast growth) under nutrient-rich conditions, but it switches to unidirectional growth and asymmetrically division (hyphal growth) under certain nutrient conditions (Sipiczki et al. 1998a, b). Upon switching to hyphal growth, the cellular organization of *Sz. japonicus* changes drastically. Cells develop large vacuoles at the non-growing tips; moreover, they accumulate granular struture at the growing tips (Furuya and Niki 2010). The rate of cell elongation increases and cytokinesis is delayed, consequently, *Sz. japonicus* forms long multi-cellular hypha during hypal growth. This switch to hyphal growth is also induced following DNA damage, and we demonstrated previously that activation of a Chk1-dependent pathway is necessary and sufficient for development of DNA damage-induced hypha (Furuya and Niki 2010).

Here, we genetically delineated the DNA damage-dependent pathway that leads to hyphal growth. Hyphae were induced via a *Sz. pombe*-like DNA damage checkpoint pathway and a Rad3^ATR^–Chk1^CHK1^-like pathway that included _SJ_ *rad3*, _SJ_ *rad26*, _SJ_ *rad1*, _SJ_ *rad9*, _SJ_ *crb2*, _SJ_ *chk1*, _SJ_ *rad24*, and _SJ_ *rad25* orthologs in *Sz. japonicus*. Interestingly, the DNA damage-dependent hyphal pathway apparently engaged in crosstalk with the nutrition-dependent hyphal pathway because cAMP inhibited DNA damage-dependent hypha, and cAMP seemed to act upstream of Chk1 kinase.

## Materials and methods

### Media

*Schizosaccharomyces japonicus* cells were cultivated as previously described (Furuya and Niki 2009). YE (yeast extract 5 g, glucose 30 g/l) was used as rich media. To induce growth of nutrient-dependent hypha, ME (malt extract 30 g, agar 20 g/l) and YEMA (Yeast extract 5 g, malt extract 30 g, glucose 10 g, agar 20 g/l) were used. A final concentration of 2 % agar was added to make solid media. CPT (camptothecin, Sigma) was used to induce DNA damage-dependent hyphae. For marker selection in YE media, 40 μg/ml of geneticin was used. EMM-2 media was used for the minimal media and the composition was reported previously in Furuya and Niki 2009.

### Strains

Strains used in this study are summarized in Table 1. Transformation of plasmids into yeast cells was performed by electroporation (Furuya and Niki 2009). Checkpoint genes in *Sz. pombe* are well-characterized, the *Sz. japonicus* orthologs of the *Sz. pombe* genes were identified by searching the database available at the Broad Institute. (http://www.broadinstitute.org//annotation/genome/schizosaccharomyces_group/MultiHome.html) (Rhind et al. 2011). These *Sz. japonicus* genes were, *crb2*; SJAG_0562, *cds1*; SJAG_04287.4, *mrc1*; SJAG_04671.4 (Furuya et al. 2012), *rad3*; SJAG_05420.4 and *rad26*; SJAG_00429.4, *rad1*; SJAG_02771.4, *tel1*; SJAG_06238.4, *rad24*; SJAG_05886.4 and *rad25*; SJAG_02576.4. The gene-disruption mutants for each of these *Sz*. *japonicus* genes were constructed as described previously (Furuya and Niki 2009).

**Table 1:** List of *Schizosaccharomyces japonicus* strains

Strains	Genotype	Source
NIG2017	h^+^ *mat*-*2017*	Furuya and Niki (2009)
NIG2028	h^−^ *mat*-*P2028*	Furuya and Niki (2009)
NIG5250	h^−^ *mat*-*P2028 chk1*-*hyp*	Furuya and Niki (2010)
NIG5439	h^−^ *mat*-*P2028 rad9::kanMX6*	Furuya and Niki (2010)
NIG5452	h^−^ *mat*-*P2028 chk1::kanMX6*	Furuya and Niki (2010)
NIG5643	h^−^ *mat*-*P2028 tel1::kanMX6*	This study
NIG5859	h^−^ *mat*-*P2028 rad1:: nat*	This study
NIG6258	h^−^ *mat*-*P2028 crb2::kanMX6*	This study
NIG6326	h^−^ *mat*-*P2028 rad26::kanMX6 spd1:: nat*	This study
NIG6362	h^−^ *mat*-*P2028 chk1*-*hyp chk1::kanMX6*	This study
NIG6402	h^−^ *mat*-*P2028 chk1*-*hyp rad9::kanMX6*	This study
NIG6437	h^−^ *mat*-*P2028 rad24::nat*	This study
NIG6443	h^−^ *mat*-*P2028 rad3::kanMX6*	This study
NIG6592	h^+^ *mat*-*2017 rad26::kanMX6*	This study
NIG6686	h^+^ *mat*-*2017 cds1::nat chk1::kanMX6*	This study
NIG6699	h^+^ *mat*-*2017 cds1::nat*	This study
NIG6701	h^+^ *mat*-*2017 mrc1::nat*	Furuya et al. (2012)
NIG6966	h^+^ *mat*-*2017 cds1::nat mrc1::nat*	This study
NIG7030	h^+^ *mat*-*2017 crb2::kanMX6 chk1::kanMX6*	This study
NIG7071	h^+^ *mat*-*2017 rad25::nat*	This study
NIG7096	h^+^ *mat*-*2017 rad24::nat chk1*-*hyp*	This study

Genotypes of *kanMX6* and *nat* indicate *kanMX6* that is G418 resistant gene, nourseothricin resistant gene

## Results

### DNA damage checkpoint pathway, but not DNA replication checkpoint pathway was required for the DNA damage-induced hypha

DNA damage-dependent hypha in *Sz. japonicus* are induced via activation of _SJ_Chk1, and disruption of the auto-inhibitory domain at the C-terminus region of _SJ_Chk1 is sufficient for the induction of hypha (Furuya and Niki 2010). However, a similar mutation in *Sz. pombe*, the cousin fission yeast, does not induce a checkpoint-dependent cell cycle delay (Tapia-Alveal et al. 2009). This phenotypic difference indicates that hyphal induction in *Sz. japonicus* is a distinct response from cell cycle delay in *Sz. pombe* and that hyphal induction in *Sz. japonicus* requires lower cellular _SJ_Chk1 activity than does cell cycle delay in *Sz*. *pombe*. Thus, we investigated whether DNA damage-dependent hyphal induction required any or all of the orthologs of the components in the DNA damage-dependent *chk1*-pathway that leads to cell cycle delay in *Sz. pombe*.

**Fig. 1:**
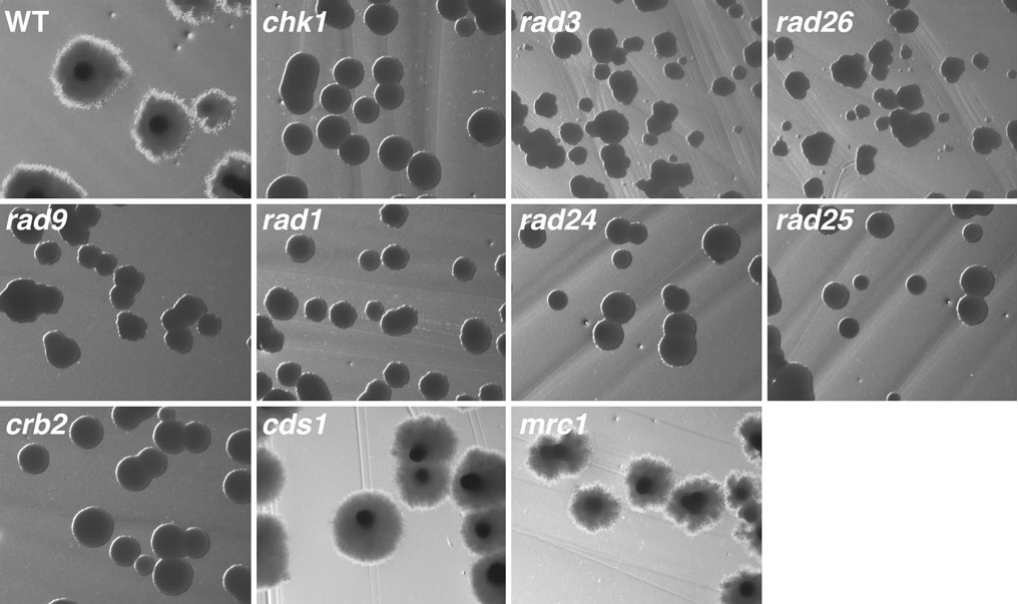
Requirement of DNA damage checkpoint genes for the DNA damage-stress-dependent hypha formation. *cds1*::*nat* and *mrc1*::*nat* colonies, but not other checkpoint mutant colonies, can present hypha when growing on YE agar media that contains camptothecin (CPT, 0.2 μM). Colonies were grown for 4 days and the photographed on the 4th day. The phenotypes of single mutant strains were summarized shown in Table 1

In *Sz. pombe*, a set of checkpoint components is required to activate _SP_ *chk1*-dependent cell cycle delay, and some of these components are assembled into distinct complexes. These components include _SP_Rad3^ATR^ and _SP_Rad26^ATRIP^, which compose the ATR kinase complex; additionally, the checkpoint clamp complex (CCC) comprises _SP_Rad9^RAD9^, _SP_Rad1^RAD1^ and _SP_Hus1^HUS1^ and functions as a DNA damage sensor complex (al-Khodairy et al. 1994; Carr 2002; Caspari et al. 2000; Edwards et al. 1999). Additionally, the mediator protein _SP_Crb2^53BP1^ is specifically required to activate _SP_Chk1^CHK1^ (Alcasabas et al. 2001; Griffiths et al. 1995; Saka et al. 1997; Tanaka and Russell 2001). A single ortholog of each of these *Sz. pombe* genes was present in the *Sz. japonicus* genome (see the “Materials and methods” section); we generated gene-disruption mutants in each of these *Sz. japonicus* genes. We also generated gene-disruption mutants in the *Sz. japonicus* orthologous of _SP_ *mrc1* and _SP_ *cds1*, which are *Sz. pombe* gene specifically involved in the DNA replication checkpoint. We then asked whether any of these *Sz. japonicus* genes were required for development of DNA damage-induced hypha. To induce hypha, cells were grown on YE agar media that contained CPT, an inhibitor of topoisomerase I, and incubated for 3 days. Wild-type cells and _SJ_ *mrc1* or _SJ_ *cds1* mutant cells formed colonies with hypha (Fig. 1). In contrast, cells carrying a _SJ_ *rad3*, _SJ_ *rad26*, _SJ_ *rad1*, _SJ_ *rad9,* or _SJ_ *crb2* mutation failed to form hypha (Fig. 1, Table 2). Thus, we concluded that the “DNA damage checkpoint genes”, but not the “DNA replication checkpoint genes”, were required for hyphal induction.

**Table 2:** *Sz. japonicus* orthologs of *Sz. pombe* checkpoint genes and hyphal induction

Genes	Gene product	Hypha in deletion mutants*
*rad* ^+^		++
*crb2*	Tudor, BRCT activating Chk1	−
*chk1*	Effector kinase for DNA damage checkpoint	−
*mrc1*	Activating Cds1	++
*cds1*	Effector kinase for DNA replication checkpoint	++
*rad1*	PCNA clamp like protein	−
*rad9*	PCNA clamp like protein	−
*rad3*	PI3-like kinase ATR ortholog	−
*rad26*	Activation of Rad3	−
*tel1*	PI3-like kinase ATM ortholog	−
*rad24*	14-3-3 protein	−
*rad25*	14-3-3 protein	±

* Hypha was assayed on 0.2 μM CPT containing agar plates

### Distinct usage of 14-3-3 genes on DNA damage-dependent hyphal pathway from DNA damage checkpoint cell cycle arrest pathway

**Fig. 2:**
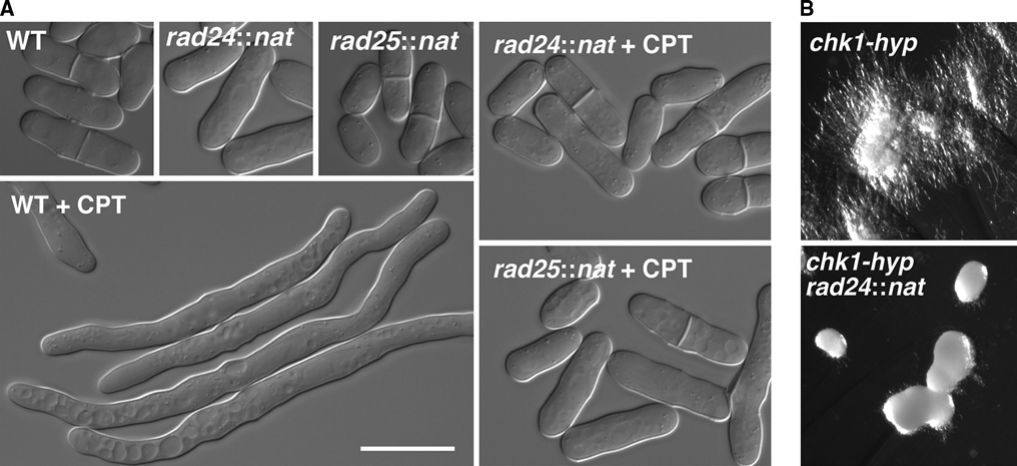
Requirement of DNA damage checkpoint genes, *rad24* or *rad25*, for the DNA damage-stress-dependent hypha formation. **a** Wild-type, *rad24*::*nat*, or *rad25*::*nat* cells were cultured in liquid YE media that contained CPT (0.2 μM) to assess hyphal induction. Cells were incubated at 30 °C for 6 h. *Scale bar*; 10 μm. **b** *chk1*-*hyp* and *chk1*-*hyp rad24*::*nat* colonies were grown at 30 °C on YE agar media to activate the *chk1*-*hyp* gene, which carried gain-of-function mutation

We extended the analysis further and examined mutations in *Sz. japonicas* orthologs of 14-3-3 proteins. 14-3-3 proteins function as homo- or hetero-dimeric complexes, and they participate in cell cycle regulation in response to DNA damage or nutritional stress and during cytokinesis (van Heusden 2009). In *Sz. pombe*, two genes—_SP_ *rad24* and _SP_ *rad25*—encode 14-3-3 proteins, and _SP_ *rad24*, but not _SP_ *rad25*, has a significant role in the DNA damage response, including in activation of the DNA damage checkpoint (Ford et al. 1994). *Sz. japonicus* also possess two 14-3-3 genes that are homologous to the *Sz. pombe* 14-3-3 genes. Perhaps interestingly, the _SJ_ *rad24*::*nat* or the _SJ_ *rad25*::*nat* mutation drastically weakened CPT-dependent hyphal induction in *Sz. japonicus*. Development of hyphal colonies was completely abolished by the _SJ_ *rad24*::*nat* mutation when cells were grown on agar plates, and it was greatly diminished by the _SJ_ *rad25*::*nat* mutation (Fig. 1). Similarly, most of the mutant cells (either _SJ_ *rad24*::*nat* or _SJ_ *rad25*::*nat* cells) grown in liquid media retained a yeast-like form, and typical hyphal morphology, such as vacuole-induction, was largely absent from these cells (Fig. 2a).

The function of _SJ_ *rad24* gene was further assessed by ectopic expression experiment of _SJ_Chk1. In *Sz. pombe*, upon DNA damage, _SP_Rad24 acts either on _SP_Chk1 or the downstream effectors of _SP_Chk1. Overexpression of the _SP_ *chk1* gene in *Sz. pombe* leads to cell death with un-attenuated checkpoint arrest, and this lethal phenotype was only compromised in _SP_ *rad24* deletion mutants, but not in other checkpoint-defective *rad* mutants (Ford et al. 1994). Here, we confirmed that _SJ_ *rad24* mutations had similar effects in *Sz. japonicus*. The expression of partially active form of _SJ_Chk1 (*chk1*-*hyp*) in *Sz. japonicus* induces hyphal growth even in the absence of genotoxic stress (Furuya and Niki 2010), and we hypothesized that the _SJ_ *rad24* deletion mutation should compromise the effect of _SJ_Chk1-Hyp activation. As expected, while a _SJ_ *chk1*-*hyp* mutant generated extensive hypha at 30 °C, a double mutant carrying _SJ_ *chk1*-*hyp* and *rad24*::*nat* mutations generated many fewer hypha than the _SJ_ *chk1*-*hyp* mutant (Fig. 2b). Notably, _SJ_ *rad9*::*kanMX6* or _SJ_ *crb2*::*kanMX6* mutations did not compromise hyphal induction in _SJ_ *chk1*-*hyp* mutants grown at 30 °C ((Furuya and Niki 2010), data not shown). Thus, the DNA damage-dependent hyphal pathway in *Sz. japonicus* was largely comparable to DNA damage checkpoint pathway in *Sz. pombe,* except that, in *Sz. japonicus*, both 14-3-3 genes (_SJ_ *rad24* and _SJ_ *rad25*) have important role in inducing hypha.

### Epistatic analysis on *Sz. japonicus* checkpoint genes

**Fig. 3:**
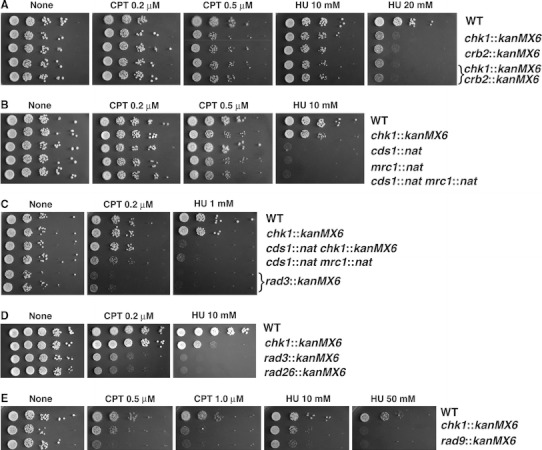
Analysis of epistatic interactions among mutants of DNA damage checkpoint genes in *Sz. japonicus*. Growth of colonies was compared under camptothecin (CPT) or hydroxyurea (HU) exposure. The *leftmost* spot contained approximately 6,000 cells when spotted; the spots to the right each represent tenfold serial dilutions; all cells were grown on YE plate that contained indicated reagents. The colonies were grown at 30 °C and photographed at 4th day. The growth of, **a** wild-type (WT) vs. *crb2*::*kanMX6*, *chk1*::*kanMX6*, *chk1*::*kanMX6 crb2*::*kanMX6* cells, **b** WT vs. *cds1*::*nat*, *mrc1*::*nat*, *cds1*::*nat mrc1*::*nat* cells, **c** WT vs. *chk1*::*kanMX6, cds1*::*nat chk1*::*kanMX6, cds1*::*nat mrc1*::*nat* and *rad3*::*kanMX6* cells, **d** WT vs. *chk1*::*kanMX6,* *rad3*::*kanMX6* and *rad26*::*kanMX6* cells, and **e** WT vs. *chk1*::*kanMX6* and *rad9*::*kanMX6* cells

We next examined cell growth upon treatment with agents that damage DNA. The *Sz. japonicus* checkpoint genes seem to have the same division of labor as do the *Sz. pombe* checkpoint genes (Table 2). Indeed, both the _SJ_ *crb2*::*kanMX6* mutants and the _SJ_ *chk1*::*kanMX6* mutants showed moderate sensitivity to hydroxyurea (HU; an inhibitor of ribonucleotide-reductase) and to CPT in colony formation assays on solid agar media (Fig. 3a). A double mutant carrying _SJ_ *crb2*::*kam* and _SJ_ *chk1*::*nat* behaved similarly to each single mutant (i.e., _SJ_ *crb2*::*kanMX6* mutants and _SJ_ *chk1*::*kanMX6* mutants); this finding indicated these two genes have mostly, if not entirely, overlapping functions.

In *Sz. pombe*, both single mutants (i.e., _SP_ *cds1*::*nat* or _SP_ *mrc1*::*nat*) and the double mutant (i.e._SP_ *cds1*::*nat* and _SP_ *mrc1*::*nat*) each showed severe sensitivity to HU; this finding indicates that these genes are each required for maintaining the integrity of the DNA replication fork (Alcasabas et al. 2001; Lindsay et al. 1998; Tanaka and Russell 2001). Cells in these three mutant *Sz. japonicus* strains showed similar colony forming abilities on plates containing HU to one another; this observation indicated that the _SJ_ *cds1* and _SJ_ *mrc1* genes function within the same pathway (Fig. 3b). In contrast, _SJ_ *mrc1*::*nat* mutants were more sensitive to CPT than were _SJ_ *cds1*::*nat* mutants (Fig. 3b); moreover, the double mutants (_SJ_ *cds1*::*nat*, _SJ_ *mrc1*::*nat* cells) were not more sensitive to CPT than were the _SJ_ *mrc1*::*nat* single mutants. This result indicated that _SJ_ *mrc1* had another role, in addition to associating with _SJ_Cds1^CHK2^ kinase, in the DNA damage response.

_SP_Rad3^ATR^–_SP_Rad26^ATRIP^ is a central *Sz. pombe* kinase complex that has a key role in both the DNA damage checkpoint and the DNA replication checkpoint. Consistent with this notion, a _SJ_ *rad3*::*kanMX6* mutation and a _SJ_ *rad26*::*kanMX6* mutation caused *Sz. japonicus* cells to be highly sensitive to CPT and to HU when cells were grown on agar media (Fig. 3c, d). The sensitivity was severer than that cause by _SJ_ *cds1*::*nat*, _SJ_ *chk1*::*kanMX6* double mutations or _SJ_ *chk1*::*kanMX6,*_SJ_ *mrc1*::*nat* double mutations (Fig. 3c). These findings indicated that, as in other eukaryotes, the _SJ_Rad3^ATR^–_SJ_Rad26^ATRIP^ complex in *Sz. japonicus* had at least one function in addition to its role in activating checkpoint effector-kinase complexes (Enoch et al. 1992; Matsuura et al. 1999). Indeed, the _SJ_ *rad3*::*kanMX6* mutants and the _SJ_ *rad26*::*kanMX6* mutants showed slow growth even without genotoxic insult. The growth defect in *Sz. pombe*_SP_ *rad3* mutants is partially suppressed by disruption of the _SP_ *spd1* gene, which is the homologue of ribonucleotide-reductase inhibitor (Liu et al. 2003; Zhao et al. 1998). In fact, introduction of the _SJ_ *spd1*::*nat* mutation into a _SJ_ *rad3*::*kanMX6* strain of *Sz. japonicus* improved cell growth; at 30 °C, the doubling time of wild-type cells was 105 min, that of _SJ_ *rad3*::*kanMX6* cells was 152 min, and that of _SJ_ *rad3*::*kanMX6 spd1*::*nat* cells was 139 min.

### Failure to keep replication fork integrity can lead to hyphal induction

**Fig. 4:**
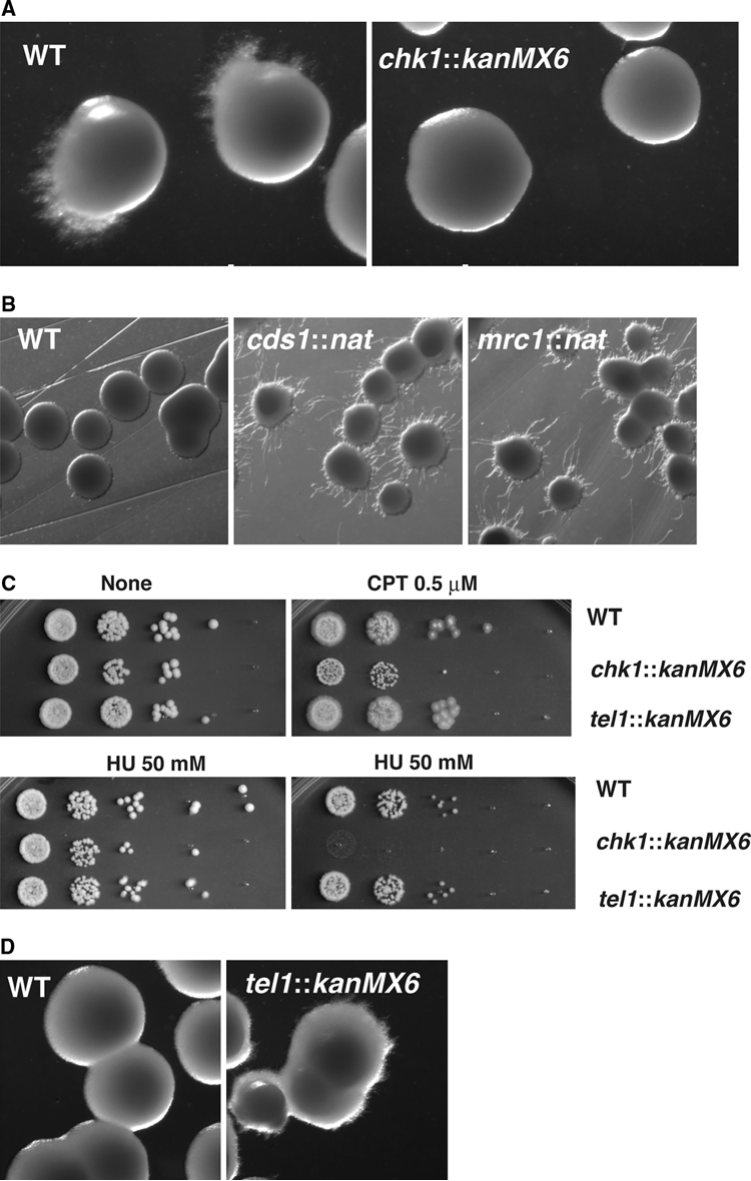
Ectopic activation of hyphal pathway in mutants of checkpoint genes. **a** Prolonged exposure to hydroxyurea HU induces hypha in wild-type colonies (WT), and this HU-mediated induction was diminished in *chk1*::*kanMX6* colonies. Cells were spread onto YE plates containing 10 mM of HU and then incubated at 30 °C for 3 days. **b** *cds1*::*nat* colonies present hypha when incubated on media containing a low concentration of HU (2 mM), but WT colonies did not. **c** Growth was compared between cells incubated on camptothecin (CPT) vs. on hydroxyurea (HU). WT, *chk1*::*kanMX6,* and *tel1*::*kanMX6* cells were compared. **d** *tel1*::*kanMX6* colonies present hypha when incubated on media containing a low concentration of HU (5 mM), but WT colonies did not

CPT causes replicative stress that can alter DNA replication fork structure (Koster et al. 2007; Ray Chaudhuri et al.). In contrast, HU reduces the cellular pool of deoxy-ribonucleotides; consequently, DNA replication forks often stall in cells treated with HU (Lindsay et al. 1998; Lopes et al. 2001). Once the integrity of the replication fork is compromised, the DNA damage checkpoint pathway is activated. Upon exposure to 10 mM HU, wild-type *Sz. japonicus* cells switched to hyphal growth (Fig. 4a), but lower HU concentrations did not cause this switch (Fig. 4a, b, d). Upon prolonged exposure to HU, replication forks may collapse and this collapse may generate DNA damage-like structures; these structures, rather than stalled replication forks, may induce the switch to hyphal growth. Indeed, HU-dependent hyphal induction was dependent on _SJ_ *chk1*, but not on _SJ_ *cds1* (Fig. 4a, b).

The integrity of stalled forks was maintained through the activity of the replication checkpoint that is governed by _SJ_Cds1^CHK2^ kinase. Consistently, the switch to hyphal growth occurred at lower HU concentrations for _SJ_ *cds1::nat* mutants than for wild-type cells (Fig. 4b); this difference was likely due to DNA damage-like structures, which were detected by _SJ_Chk1, that resulted from DNA replication fork collapse in the mutants, but not in the wild-type cells (Boddy et al. 1998; Lindsay et al. 1998).

HU-induced hyphae were also elicited via a defect in _SJ_Tel1^ATM^. The *tel1* genes in yeasts encode kinases homologous to Rad3^ATR^. In yeasts, unlike Rad3^ATR^, which has a major role in the DNA damage response, Tel1^ATM^ has a minor contribution to the resistance to DNA damage. However, Tel1^ATM^ can phosphorylate various checkpoint proteins, and it is involved in double-stranded DNA break repair; these observations indicate that Tel1^ATM^ may participate in efficient DNA damage response (D’Amours and Jackson 2001; Furuya et al. 2004; Nakada et al. 2003; Usui et al. 2001; Zhao et al. 2003). The *Sz. japonicus* ortholog of _SJ_ *tel1* was deleted, and these mutant cells were tested for sensitivity to HU and to CPT. The _SJ_ *tel1*::*kanMX6* mutants did not show obvious sensitivity to HU or CPT (Fig. 4c). However, the mutants did exhibit ectopic induction of hypha at the lower concentration of HU (5 mM) (Fig. 4d); this observation indicated that *Sz. japonicus*_SJ_ *tel1* has a role in genome maintenance at stressed replication forks.

### Crosstalk between DNA damage-dependent and nutritional stress-dependent hyphal regulation

**Fig. 5:**
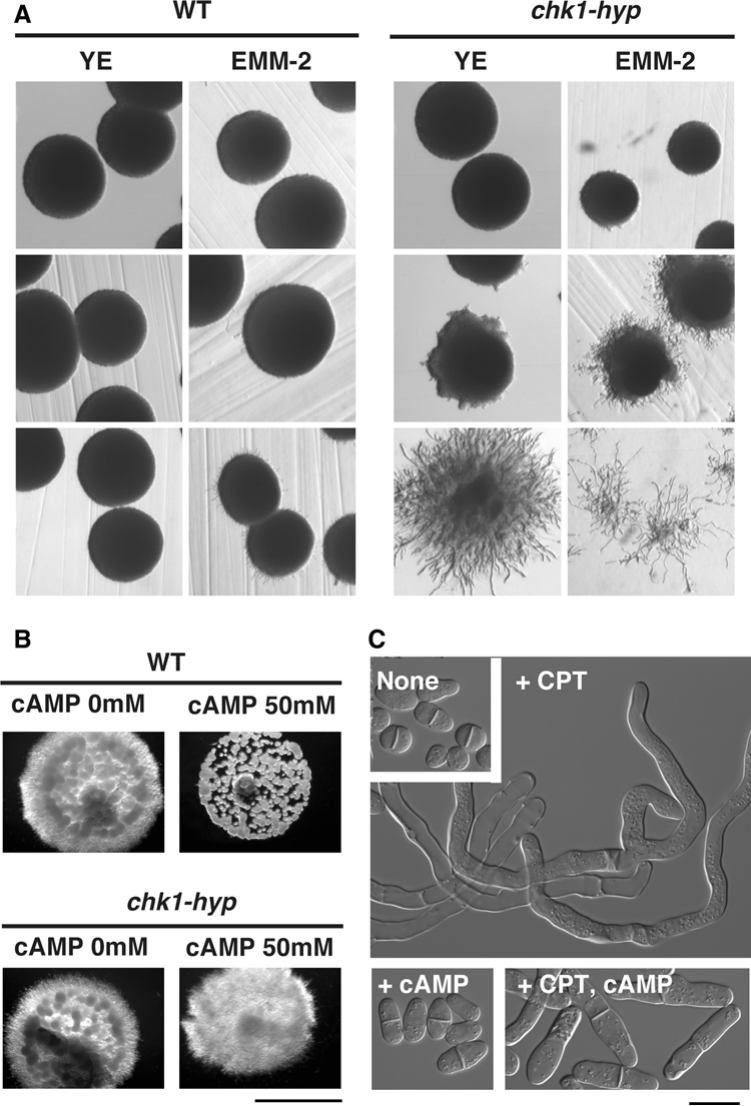
The nutrient stress signal could affect DNA damage-dependent hypha. **a** Hyphal formation in *chk1*-*hyp* mutants was compared between cells grown on YE vs. EMM-2 media. On YE agar media, *chk1*-*hyp* induced hypha at 30 °C, but on EMM-2 agar media, the transgene induced hypha at 33 °C. The colonies were grown at the indicated temperature for 3 days and then photographed on the 3rd day. **b** cAMP inhibited CPT-induced hypha formation, but did not affect *chk1*-*hyp* dependent hypha on agar media (*bar*; 5 mm) **c** cAMP (50 mM) inhibited CPT-induced hypha formation in wild-type cells grown in liquid media (*bar*; 10 μm). 0.2 μM of CPT was used

DNA damage stress and nutritional stress both induce hyphal differentiation. The cellular morphology of hypha induced by DNA damage stress was indistinguishable from that of hypha induced by nutritional stress. Thus, we assumed that the signals derived from the different stress responses could converge onto the same hyphal regulator. If so, these two stress responses (i.e., the DNA damage stress response and the nutritional stress response) could affect hyphal induction synergistically. Therefore, we compared temperature-dependent hyphal induction in _SJ_ *chk1*-*hyp* mutants under several nutrient conditions. The _SJ_ *chk1*-*hyp* mutants induced hypha at low temperatures even in the absence of DNA damage stress; moreover, when grown on the nutrient-rich agar media (YE media), the mutants formed hyphal colonies at 30 °C (Fig. 5a). Indeed, _SJ_Chk1-dependent hyphal induction was enhanced when the mutants were grown on EMM-2 medium at 30 *°*C; these growth conditions impose nutrient stress. Furthermore, on the nutrient-poor media (EMM-2), _SJ_ *chk1*-*hyp* mutant could induce hypha at a higher temperature; 33 °C (Fig. 5a). Hyphal colonies usually invade the agar and become resistant to being washed off plates by flowing water (Furuya and Niki 2010) (Supplementary Figure 1). As expected, when _SJ_ *chk1*-*hyp* cells were spotted and incubated on EMM-2 agar versus YE agar media, more cells remained in the EMM-2 agar after plates were washed with flowing water.

### cAMP diminished CPT-induced hypha, but not *chk-hyp* induced hypha

Induction of hypha by _SJ_ *chk1*-was enhanced when _SJ_ *chk1*-*hyp* cells were switched from YE medium to EMM-2 medium. In *Sz. pombe*, switching from YE medium to EMM-2 medium correlates with the repression of cAMP-dependent signaling (Yamashita et al. 1996). Thus, we speculated that an increase in the concentration of cellular cAMP could inhibit hyphal induction. Indeed, CPT-induced hypha was inhibited by 50 mM of cAMP (Fig. 5b, c). Reportedly, cAMP reverts nutrient-dependent hyphal growth to yeast growth (Sipiczki et al. 1998b). Thus, we initially thought that the common hypha-regulator that could sit downstream of both DNA damage- and nutrient-stress signaling was repressed by cAMP. However, perhaps surprisingly, cAMP did not inhibit _SJ_ *chk*-*hyp* induced hypha (Fig. 5b); this finding indicated that cAMP could act at upstream of _SJ_Chk1.

## Discussion

**Fig. 6:**
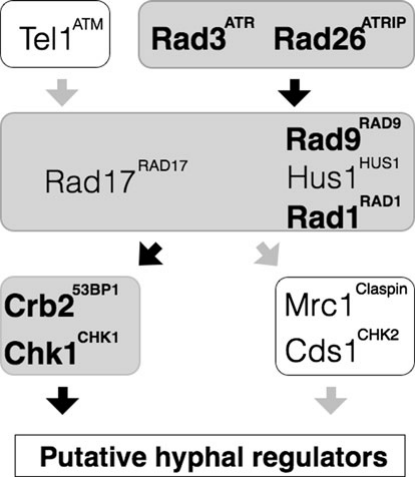
The checkpoint-dependent hyphal pathway. The *diagram* summarizes the functional relationship between genes involved in stress response and hyphal induction in *Sz. japonicus* and DNA damage checkpoint in *Sz. pombe*. The groups of proteins involved in hyphal induction are shaded in *gray* and the pathways to activate hypha are indicated by *black arrows*. The genes indicated in *bold* have been shown to be required in DNA damage-dependent hyphal induction (this study or Furuya and Niki 2010). Those orthologous genes involved in DNA damage hypha are required in DNA damage checkpoint in *Sz. pombe*

In this report, we delineated the DNA damage-dependent hyphal pathway in *Sz. japonicus,* an organism that is included in the fission yeast genus. Based on genomic sequencing information, we know that *Sz. japonicus* has a set of genes that are orthologous to the *Sz. pombe* genes that are involved in checkpoint responses (Fig. 6). However, we have previously shown that, in *Sz. japonicus,* activation of this checkpoint led to a cell fate different from the cell fate adopted by *Sz. pombe* cells; upon activation of this checkpoint, *Sz. japonicus* cells begin hyphal differentiation, but *Sz. pombe* cells enter a cell cycle delay. Importantly, in *Sz. japonicus*, DNA damage checkpoint-dependent hyphal induction seemed to require lower amount of DNA damage than DNA damage-induced cell cycle delay. This fact prompted us to investigate the precise division of labor among checkpoint genes upon hyphal differentiation. Furthermore, since hypha is also induced upon nutrient changes, we tested whether these hypha pathways, which are activated by different stimuli, could engage in crosstalk (Sipiczki et al. 1998b).

### DNA damage checkpoint pathway in *Sz. pombe* is equivalent to DNA damage hyphal pathway in *Sz. japonicus*

We compared the division of labor among the checkpoint genes by constructing gene-disruption mutants for checkpoint genes and asked whether the genes are required for DNA damage-dependent hyphal differentiation. DNA damaged-induced hyphal differentiation in *Sz. japonicus* required _SJ_ *rad3* ^*ATR*^, _SJ_ *rad26* ^*ATRIP*^, _SJ_ *rad9* ^*Rad9*^, _SJ_ *rad1* ^*Rad1*^, _SJ_ *crb2* ^*53BP1*^, _SJ_ *chk1* ^*CHK1*^, _SJ_ *rad24* and _SJ_ *rad25*. These genes are the *Sz. japonicus* counterparts of genes that encode proteins in the DNA damage checkpoint pathway of *Sz. pombe*. In contrast, *Sz. japonicus*_SJ_ *mrc1* ^*Claspin*^ and _SJ_ *cds1* ^*CHK2*^, the counterparts of components of the *Sz. pombe* DNA replication checkpoint, were not required for hyphal differentiation. Thus, *Sz. japonicus* had the same series of checkpoint components as does *Sz. pombe* (Carr 2002). In other words, the DNA damage checkpoint pathway in *Sz. pombe* corresponded to the DNA damage hyphal pathway in *Sz. japonicus*.

### Cooperation of two 14-3-3 genes at hyphal induction

In fission yeast, two genes (*rad24* and *rad25*) encode 14-3-3 proteins. In *Sz. pombe*, induction of the DNA damage-dependent checkpoint is mainly dependent on one 14-3-3 gene, _SP_ *rad24*. However, *Sz. pombe* cells with a _SP_ *rad24* null mutation do undergo partial checkpoint-induced arrest; therefore, _SP_ *rad25* might have a partially overlapping role in this checkpoint. Originally in *Sz. pombe*, _SP_ *rad25* was isolated as a multi-copy suppressor of a _SP_ *rad24* deletion mutant. Although a single deletion mutant of _SP_ *rad25* is not defective in DNA damage response, it is synthetic lethal with _SP_ *rad24* deletion mutant (Ford et al. 1994). Interestingly, we found that the *Sz. japonicus*_SJ_ *rad24* and _SJ_ *rad25* genes both had a role in the induction of DNA damage-dependent hypha. The _SJ_ *rad24* deletion mutant completely abolished hyphal induction when *Sz. japonicus* cells were grown on agar plate. However, colonies from the _SJ_ *rad25* deletion mutant strain seemed to have a residual, but greatly diminished, ability to form the hypha when exposed to DNA damaging agents. Furthermore, few of the _SJ_ *rad24*-null cells or the _SJ_ *rad25*-null cells developed typical hyphae with an elongated, vacuole-rich morphology in liquid culture. In *Sz. japonicus*, the two 14-3-3 proteins could act on different steps of hyphal induction and concomitant regulation may enable the cells to form multi-cellular hypha upon DNA damage response.

Utilization of each 14-3-3 protein is likely to depend on context. Wild-type *Sz. japonicus* cells form hypha under prolonged incubation on agar media where nutrients are limited. In this study, we used a synthetic minimal media, EMM-2. In this case, hypha development was diminished for _SJ_ *rad25* deletion mutants, but not for _SJ_ *rad24* mutants (Supplementary Figure 2A). In contrast, when we used YEMA, where malt extract was a main carbon source, hyphae were formed efficiently even with a _SJ_ *rad24* or _SJ_ *rad25* deletion mutation (Supplementary Figure 2B). Thus, signals from different stress seems to use different combination of 14-3-3 proteins to reach the hypha-regulator.

14-3-3 proteins bind preferentially to phospho-peptides; thus, 14-3-3 proteins often influence the function of phosphorylated proteins; for example, a 14-3-3 protein can cause a phosphorylated protein to relocate or to increase or decrease protein–protein interactions and thereby adopt a new function. 14-3-3 proteins can bind to many proteins; in case of budding yeast, 4 % of proteins in the cell are potential target of 14-3-3 proteins, and these potential are involved in various aspects of many cellular activities (van Heusden 2009). Some of the known activities that 14-3-3 proteins participate in are (1) activating DNA damage checkpoint, (2) delaying cytokinesis, and (3) promoting sexual development signal (Ford et al. 1994; Kitamura et al. 2001; Mishra et al. 2005); moreover, manipulation of these activities upon extracellular stress can cause cells to adopt the morphology of hyphal cells (i.e., the cells elongate and remain attached even after the completion of septation). Searching for specific targets of each *Sz. japonicus* 14-3-3 protein may uncover the molecular basis of different hyphal pathways.

### Crosstalks between nutrient-stress pathways

Both DNA damage stress and nutrient stress induce hypha in *Sz. japonicus*. Nutrient stress induced hyphal cells were morphologically indistinguishable under a microscope from those induced by DNA damage. Thus, we speculated that these two pathways might converge onto one regulator of hyphal differentiation. We, at least, speculate that the two pathways might share same components or engage in crosstalk. Here, we demonstrated that nutrient stress could enhance DNA damage-induced hyphal differentiation. Additionally, we presented that cAMP might be a key second messenger in the control of both hyphal induction pathways. Induction of hypha upon nutrient stress is repressed by addition of cAMP, and we showed here that induction of hypha following DNA damage was also inhibited by addition of cAMP. Perhaps interestingly, hypha induced via introduction of an active form of _SJ_ *chk1* were not inhibited by cAMP, and this finding may have indicated that cAMP could act upstream of _SJ_ *chk1*. At present, we do not know how cAMP could affect the DNA damage response; cAMP may affect chromatin regulation that in turn affects global transcription, or cAMP regulation may directly affect the activity of checkpoint proteins.

cAMP level is known to be upregulated under glucose-enriched conditions in eukaryotic cells (Broach 1991; Thevelein 1994). In case of *Sz. pombe*, synthetic media like EMM-2 could correlate with the downregulation of the cAMP pathway (Yamashita et al. 1996). We speculate that cAMP level could tune the extent of the checkpoint-dependent hyphal differentiation, and we believe that mechanism behind this may involve the molecular crosstalk between two different extracellular stresses; nutrient stress and DNA damage stress.

## Conclusion

Here we showed that in *Sz. japonicus* DNA damage triggers cellular differentiation utilizing the same set of DNA damage checkpoint genes as used by *Sz. pombe* to promote a cell cycle arrest. In addition, slight differences in the involvement of the cAMP pathway could lead to new insights on the relationship between DNA damage and nutrient stress sensing in these yeasts.

## Electronic supplementary material

Below is the link to the electronic supplementary material.

**Supplementary Figure 1** Hypha that were induced by the *chk1-hyp* mutation become more resistant to washing when grown on EMM-2 agar media. Wild-type (WT) or *chk1-hyp* cells were spotted onto YE or EMM-2 agar media and incubated for 4 days at the respective temperatures. After the colony growth, agar plates were washed with flowing water. (PDF 79 kb)

**Supplementary Figure 2** Wild type or *rad24*::*nat or rad25*::*nat* cells were spotted on **A.** EMM-2 or **B**. YEMA agar media; the ability these cells to form hypha was assessed. Plates were incubated at 33 ℃ for 5 days and then photographed on the 5th day. (PDF 47 kb)
