# Supplementary figures and images for: Hyphal differentiation induced via a DNA damage checkpoint-dependent pathway engaged in crosstalk with nutrient stress signaling in Schizosaccharomyces japonicus
Source: Curr Genet. 2012 Oct 23;58(5):291–303. doi: 10.1007/s00294-012-0384-4 (PMC3490063; doi:10.1007/s00294-012-0384-4)

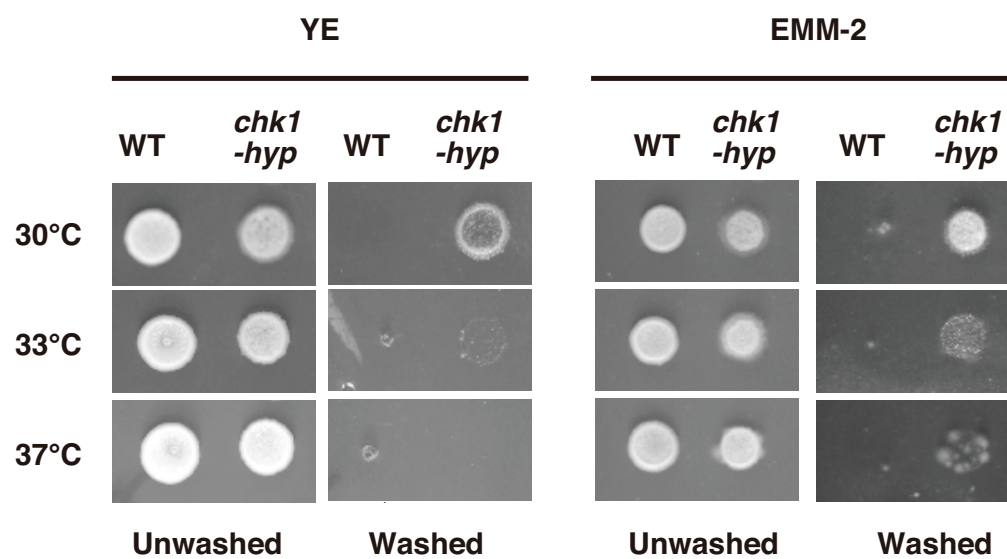

Furuya & NIKI, Supplementary Fig. 1

Supplement: Supplementary file 1 — Supplementary Figure 1 Hypha that were induced by the chk1-hyp mutation become more resistant to washing when grown on EMM-2 agar media. Wild-type (WT) or chk1-hyp cells were spotted onto YE or EMM-2 agar media and incubated for 4 days at the respective temperatures. After the colony growth, agar plates were washed with flowing water. (PDF 79 kb) [file 294_2012_384_MOESM1_ESM.pdf]

**A**

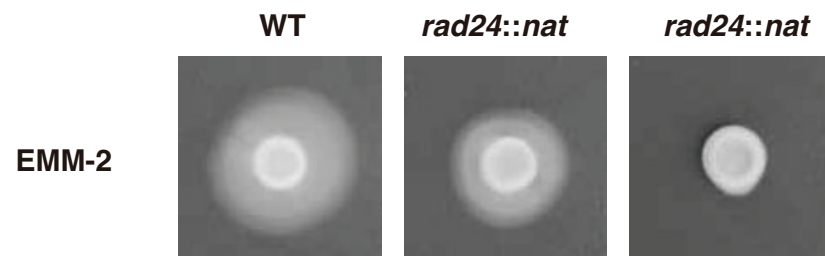

**B**

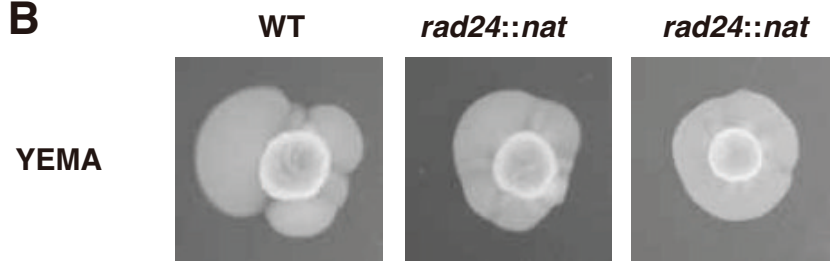

Supplement: Supplementary file 2 — Supplementary Figure 2 Wild type or rad24::nat or rad25::nat cells were spotted on A. EMM-2 or B. YEMA agar media; the ability these cells to form hypha was assessed. Plates were incubated at 33 ℃ for 5 days and then photographed on the 5th day. (PDF 47 kb) [file 294_2012_384_MOESM2_ESM.pdf]
